# A Longitudinal Study with a Laser Methane Detector (LMD) Highlighting Lactation Cycle-Related Differences in Methane Emissions from Dairy Cows

**DOI:** 10.3390/ani13060974

**Published:** 2023-03-08

**Authors:** Ana Margarida Pereira, Pedro Peixoto, Henrique J. D. Rosa, Carlos Vouzela, João S. Madruga, Alfredo E. S. Borba

**Affiliations:** Faculdade de Ciências Agrárias e do Ambiente, Instituto de Investigação em Tecnologias Agrárias e do Ambiente (IITAA), Universidade dos Açores, Campus de Angra do Heroísmo, Rua Capitão João d’Ávila, 9700-042 Angra do Heroísmo, Portugalhenrique.jd.rosa@uac.pt (H.J.D.R.); carlos.fm.vouzela@uac.pt (C.V.); joao.s.madruga@uac.pt (J.S.M.); alfredo.es.borba@uac.pt (A.E.S.B.)

**Keywords:** Azores, dairy cows, extensive farming systems, laser methane detector (LMD), methane, milk sustainability

## Abstract

**Simple Summary:**

Within anthropogenic sources, agriculture contributes the most to greenhouse gas (GHG) emissions. The on-field assessment of methane emissions from livestock is crucial for testing and implementing mitigation strategies to reduce the deleterious effects of GHG on climate. Therefore, this article presents a longitudinal study in which measurements of enteric methane emissions from dairy cows (Jersey and Holstein-Freisian raised under a semi-extensive management system) were performed with a tool previously described as reliable, portable, and cost-effective: the laser methane detector (LMD). The results showed breed-related differences in methane emissions when milk yield was not considered, challenging us to rethink milk traits and breeding choices. Moreover, methane emissions were lower in cows in early lactation as well as in primiparous, likely reflecting concentrate supplementation and energy metabolism. The study pointed out the opportunity to design and test regional tailored mitigation strategies, including nutritional management, for higher methane emission periods within cows’ lactation cycle, while ensuring the enhancement of productivity.

**Abstract:**

Reversing climate change requires broad, cohesive, and strategic plans for the mitigation of greenhouse gas emissions from animal farming. The implementation and evaluation of such plans demand accurate and accessible methods for monitoring on-field CH_4_ concentration in eructating breath. Therefore, this paper describes a longitudinal study over six months, aiming to test a protocol using a laser methane detector (LMD) to monitor CH_4_ emissions in semi-extensive dairy farm systems. Over 10 time points, CH_4_ measurements were performed in dry (late gestation) and lactating cows at an Azorean dairy farm. Methane traits including CH_4_ concentration related to eructation (E_CH4) and respiration (R_CH_4_), and eructation events, were automatically computed from CH_4_ measured values using algorithms created for peak detection and analysis. Daily CH_4_ emission was estimated from each profile’s mean CH_4_ concentration (MEAN_CH_4_). Data were analyzed using a linear mixed model, including breed, lactation stage, and parity as fixed effects, and cow (subject) and time point as random effects. The results showed that Holsteins had higher E_CH_4_ than Jersey cows (*p* < 0.001). Although a breed-related trend was found in daily CH_4_ emission (*p* = 0.060), it was not significant when normalized to daily milk yield (*p* > 0.05). Methane emissions were lower in dry than in lactation cows (*p* < 0.05) and increased with the advancement of the lactation, even when normalizing it to daily milk yield (*p* < 0.05). Primiparous cows had lower daily CH_4_ emissions related to R_ CH_4_ compared to multiparous (*p* < 0.001). This allowed the identification of periods of higher CH_4_ emissions within the milk production cycle of dairy cows, and thus, the opportunity to tailor mitigation strategies accordingly.

## 1. Introduction

The climate-neutral economy to be achieved by 2050 requires substantial cuts in greenhouse gas (GHG) emissions in several sectors, including agriculture. Within agriculture, the most relevant sources of GHG are agricultural soils and enteric fermentation, with cattle standing as the most contributing livestock species [1]. Reducing animal farming GHG emissions requires broad, cohesive, and strategic plans. However, the diversity of animal farming systems around the globe, associated with their socioeconomic and cultural contexts, creates different GHG emissions patterns, which require regionally tailored measures for emissions reduction [2]. Several years of research on CH_4_ mitigation strategies have shown promising results through genetics [3], nutritional management [4], and vaccination [5], mostly in the context of experimental studies. Nevertheless, a critical target in this search is the suitability of techniques for measuring livestock CH_4_ emissions and a likely limiting factor for broadening the assessment at larger scales. While the low accuracy of indirect measurement techniques such as in vitro incubation limits their use for screening assays [6], highly accurate direct continuous measurement techniques such as “the gold standard” respiratory chamber are mainly limited by their high cost and the need for single animal confinement [7]. Similarly, other direct measurement techniques—either continuous, such as Sulfur Hexafluoride (SF_6_) [8], or spot-sampling-based, such as GreenFeed^®^ [9]—likewise involve high costs, despite retrieving good estimations of daily CH_4_ emissions with minimal disturbance of animals’ routines [10]. With a higher risk of compromised repeatability but substantially less associated costs, other spot-sampling-based techniques, such as laser methane detection (LMD), non-dispersive infrared, and Fourier-transformed infrared breath analyzers, have proved reasonable for on-field application [11,12,13,14]. In addition to accuracy, costs, portability, throughput, operating convenience, and personal expertise stand out as critical appropriateness criteria for CH_4_ measurement methods. 

The LMD is a handheld laser detector based on infrared absorption technology, featuring both a visible (green color for guiding purposes) and an invisible laser beam with a wavelength that CH_4_ absorbs, allowing determination of the amount absorbed through the analysis of the diffuse laser that is reflected back to the instrument. The measured gas volume is expressed by the CH_4_ column density (ppm-m), a unit meaning CH_4_ density (ppm) multiplied by the width (m). The specifications of the instrument imply a certain susceptibility to environmental conditions and knowledge of the limitations of the outcomes [15]. The main advantages of LMD are portability, ease of operation, and low purchase and running costs, in contrast with the disadvantages of medium throughput and high labor [7]. From an animal welfare point of view, the pros of LMD are the non-invasiveness, despite some restraint that might be required to hold animals in position, especially when measuring grazing livestock. 

This paper describes a longitudinal study over six months testing the suitability of an LMD to monitor CH_4_ emissions in semi-extensive farm systems. To achieve this, the concentration of CH_4_ in the exhaled airflow (breathing and eructation) of dairy cows while progressing in the lactation cycle was measured, with the aim of identifying the effects related to the breed, parity, and stage of lactation. 

## 2. Materials and Methods

This study was conducted from February to July 2022 at the Experimental Dairy Farm Unit of the School of Agrarian & Environmental Sciences of the University of the Azores, located in a middle altitude region of Terceira Island (390 m above the sea; latitude 38°41′52.8″ N; longitude 27°10′24.6″ W). The climate of the region is classified as humid mesothermal with oceanic characteristics, with annual rainfall varying from 1800 to 2200 mm, mean annual temperatures of 14.5 °C, and annual relative humidity varying from 88 to 92% [16]. 

The farm is representative of traditional Azorean dairy cattle farming, with an area of 55 ha and a herd size of around 80 heads including 60 dairy cows (animal units/ha ≈ 1.5), under a rotational grazing system throughout the year in permanent pasture. The mean annual milk production of the farm in the last 5 years was ≈ 400,000 L. The percentage of lactating cows in the 1st, 2nd, 3rd, 4th, and 5th lactation was, respectively, 32, 26, 27, 9, and 6%. The daily milk production and total milk production (305-d) of each cow were obtained from the dairy herd management software (Alpro, DeLaval, Shrewsbury, UK). The daily milk production was the average of 7 days, overlapping CH_4_ measurement time points. Data on each cow’s fat and protein milk content were analyzed monthly by an official laboratory (SERCLA, Angra do Heroísmo, Portugal), using the Milkoscan^™^ method. 

Lactating cows and cows in late gestation undergoing acclimatization to milking routines were included in the study. The recruited cows had a breed distribution of 23% Jersey and 77% Holstein-Friesian, and an average of 51 ± 18.5 months of age (max 90; min 14). Further detail on the recruited cows at each time point is available as Supplementary Material (Table S1). All cows kept their normal routine of moving from the assigned grazing paddocks to the milking parlor twice a day (6 am and 4 pm). Minimal disturbance of the animals’ milking routine on measurement days was guaranteed to avoid stressing cows. 

In addition to voluntary fresh grass intake, a concentrate was supplied at the time of milking. The chemical composition of the concentrate and the corresponding methodology of analysis are provided as Supplementary Material (Table S2). The daily amount of concentrate given was 250 g per L of milk to primiparous cows during all lactation, and to multiparous at the early stage of lactation, whereas all the other cows received 200 g of concentrate per L of milk. Water was permanently available.

**Figure 1:**
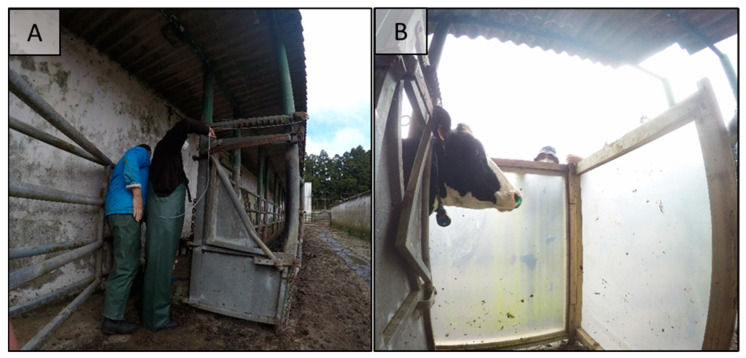
The actual image of the place where LMD was operated, captured with a wide-angle lens. (**A**) Handling race with a cattle head holder at the end for cattle contention; (**B**) positioning of wind protection panels during an LMD measurement (laser is visible as a green dot in the right nostril).

Methane emitted through eructation and respiration was measured at 10 time points using an LMD (LaserMethaneMini^™^ Tokyo Gas Engineering, Tokyo, Japan). After evening milking, cows leaving the parlor were routed to a handling race provided with a cattle head holder, where the CH_4_ measurements took place. The design of the handling race allowed partial wind protection, due to a roof and side wall located behind the LMD operator. Two panels were placed at the left and frontal positions of the animals’ head, obliterating airflow from the open zone (Figure 1). The LMD was pointed to the cows’ right nostrils at a fixed distance of 1.2 m. The length of measurement was 120 s, with a sampling rate of 0.5 s. A CH_4_ background environment was obtained in the exact same spot measurement without the animals. The same operator took all measurements. Data were transmitted to an Android device running the GasViewer app (Tokyo Gas Engineering) via Bluetooth connection, producing a separate CSV file per measurement, which was analyzed offline. 

**Figure 2:**
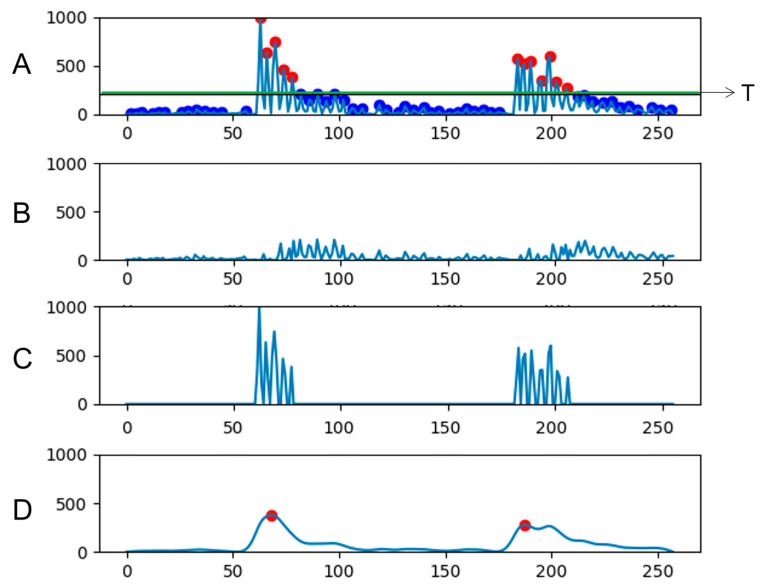
Graphical plot of a profile: y and x axis represent, respectively, CH_4_ measured values (ppm-m) and length of measurement (s). (**A**) the signal plot with all identified CH_4_ peaks: red dots and blue dots correspond, respectively, to eructation and respiratory CH_4_ peaks, T indicates the threshold; (**B**) plot of CH_4_ respiratory peaks; (**C**) plot of CH_4_ eructation peaks; (**D**) plot of eructation events.

A series of algorithms were created to collate individual files containing the measured CH_4_ values (signal), identify peaks, and compute the CH_4_ traits of each cow at a single time point (profiles), using Python 3.10. Figure 2 depicts one CH_4_ profile and the sequence of calculations to obtain the CH_4_ traits. First, potential artifacts were removed by applying a cap value of 1000 to 481 out of ≈ 130,000 CH_4_ measured values (unphysiological high). Second, the arithmetic mean (μ) and standard deviation (σ) of the signal within a profile (Figure 2A) were calculated. Third, a threshold (T) for each profile was defined as T=μ+σ. The signal was then divided into a respiratory (Figure 2B) and eructation (Figure 2C) component, based on the T. The respiratory and eructation signals were then processed to allow the identification of respiratory and eructation peaks, based on their prominence (simply, how much the peak stands out due to its intrinsic height, and its location relative to another peak). The division into components enabled the identification of eructation events and the estimation of respiratory rate. The respiratory rate was estimated from the respiratory peaks. For the identification of eructation events, a low-pass filter (2nd order Butterworth filter) was applied to soothe the signal and remove the short-term fluctuations, leaving the longer term trend (Figure 2D). The methane traits of each profile included the MEAN_CH_4_ (arithmetic mean of all peak values), E_CH_4_ (arithmetic mean of eructation peak values), and R_CH_4_ (arithmetic mean of respiratory peak values). Complementarily, the percent shares of eructation and respiratory emissions were estimated from the sum of CH_4_ eructation peak values (SUM_E_CH_4_) and the sum of respiratory peak values (SUM_R_CH_4_) divided, respectively, by the sum of all CH_4_ peak values (SUM_CH_4_). 

The amount of CH4 emitted per day was estimated using the following equation proposed by Lanzoni et al. (2022) [17]:

(1) $$CH_{4}\;\left(g\;day^{-1}\right)=MEAN_{CH_{4}}\times V\times R\times \alpha \times \beta \times 10^{-6}\times 1440$$

in which V is the tidal volume (3800 mL), R is the respiratory rate (respiratory peaks), α is the conversion factor of CH_4_ production from mL to g (0.000667 g/mL), β is the correction factor for the difference between breath and total CH_4_ production (10). The estimation of daily CH_4_ emission was normalized to the daily milk production of each cow. 

Data were analyzed using the mixed linear model procedure of SAS^®^ Studio software, LTS 3.81(2022), (SAS^®^ OnDemand for Academics, NC, USA) that employs restricted maximum likelihood as the estimation method. The following model was used for data analysis:

(2) $$y_{ijklm}=x+B_{i}+LS_{j}+LN_{k}+C_{l}+t_{m}+\varepsilon_{ijklm}$$

in which y is either the milk or CH_4_ trait, x is the intercept, B is the fixed effect of breed (i = Jersey, Holstein-Friesian), LS the fixed effect of lactation stage (j = dry, early, mid, and late), LN is the fixed effect of parity (k = 1, 2, 3, and ≥4), C is the random effect of subject cow, t is the random effect of time point, and ε is the random error. The Kenward–Roger method was used to compute denominator degrees of freedom [18]. The least-square means (lsmeans) of the fixed effects were computed based on the linear model, and multiple comparisons were performed using a Tukey–Kramer test. The statistical level of significance and trend was considered when *p* < 0.05 and *p* < 0.1, respectively.

## 3. Results

A total of 460 CH_4_ profiles were obtained from 55 dairy cows collected in 10 time points over 6 months (the number of cows in each data point varied). Table 1 shows the descriptive statistics of all CH_4_ profile variables, calculated from raw LMD data. The lowest CH_4_ measured values of profiles ranged from 0 to 14 ppm, whereas the maximum ranged from 108 to 6170 ppm. The frequency of 0 was 6 per 1000 measured values, equitably distributed across profiles. A total of 170 profiles had at least one peak above 1000 ppm-m. The recalculated MEAN_CH_4_, assuming a 1000 cap for maximum values, retrieved a MEAN_CH_4_ of 81 ± 30.1 ppm. The MEAN_CH_4_ reflected both the concentration of CH_4_ in breath from eructation and respiration events, in an average proportion of 53:47%, with the maximum share of eructation and the lowest share of respiration, respectively, at 63 and 37%. The CH_4_ background environment varied from 0 to 5 ppm-m (2 ± 1.3 ppm-m). 

The effect of breed, lactation stage, and parity on milk and CH_4_ traits are displayed in Table 2. Daily milk production was significantly higher in Holstein-Friesian compared to Jersey cows (*p* < 0.01), contrary to protein and fat milk content, which were higher in Jersey (*p* < 0.01). Concerning the lactation stage, milk production decreased significantly from early to late lactation (*p* < 0.01), whereas protein and fat milk content increased in late lactation compared to both the early and mid-stages (*p* < 0.01). Primiparous cows produced less milk than multiparous (*p* < 0.01). The milk yield of cows in the fourth lactation stage and over was lower than that of cows in the second and third lactations (*p* < 0.01). The fat content of milk was not affected by parity (*p* > 0.05), but protein content was higher in primiparous than multiparous cows (*p* < 0.01). Breed-related differences were observed in E_CH_4_ (*p* < 0.01), being higher in Holstein-Friesian than Jersey cows, with R_CH_4_ tending to follow the same pattern (*p* = 0.059). The estimation of daily CH_4_ emitted also tended to be higher in Holstein-Friesian than in Jersey cows (*p* = 0.060); however, when normalizing to milk yield, no breed-related differences were found (*p* > 0.05). E_CH_4_ and R_CH_4_ were significantly lower in dry cows compared to cows in lactation (*p* < 0.01). Early lactation cows had higher E_CH_4_, R_CH_4_, and daily CH_4_ emissions than cows in the mid and late lactation stages (*p* < 0.01), even when normalizing to milk production (*p* < 0.01). The number of eructation events was not affected by breed, lactation stage, or parity (*p* > 0.05). Finally, the results showed that primiparous cows emitted less CH_4_ (E_CH_4_ *p* = 0.074; R_CH_4_ *p* < 0.01) compared to multiparous, even when normalizing to milk yield (*p* < 0.05). 

## 4. Discussion

To our best knowledge, this article is the first to describe livestock in vivo measurements of CH_4_ emission in the Azores. This is important because livestock represents a great share of the economy, strongly implicated in the history, culture, and social organization of all nine Azorean islands. Indeed, the ratio of cattle to the human population in the Azores equals 1:1.2, with a dairy cow population of ≈92 thousand and an annual milk production of ≈652 million L [19]. The production system is predominantly semi-extensive with permanent pastures that represent 43% of the region’s land area [19]. The end of European milk quotas, as well as the declining interest and lack of financial incentive for younger generations to invest in agriculture, forces bringing about change in the sector once marked by small-sized family-owned businesses, whose profitability was mainly due to low mechanization, labor, and infrastructures [20]. We are now witnessing a decrease in the number of farms offset by an increase in farm size, and a search for greater efficiency and productivity to cope with a competitive market [21]. Yet, shaping of the dairy farming sector can no longer be achieved without considering both economic and environmental sustainability, the latter being inherently linked to GHG emissions. 

The mean, respiratory, and eructation CH_4_ concentrations of all profiles were consistent with previous LMD-based studies performed in dairy cows, despite the unavoidable differences related to the measurement conditions. Sorg et al. (2018) reported mean and eructation CH_4_ concentrations of, respectively, 97 ± 44 and 350 ± 148 ppm-m, operating LMD in a free-stall dairy barn with partially open walls and a 2.5 m measurement distance [13]. In another work, Kobayashi et al. (2021) estimated ≈ 66 g of daily CH_4_ emissions in indoor-fed Fogera dairy cows [22]. Pinto et al. (2020) found mean, respiratory, and eructation CH_4_ concentrations of, respectively, 43 ± 34.9, 16 ± 17.2, and 108 ± 148.3 ppm [23]. The values reported by Pinto et al. (2020) are lower than the ones herein reported. Yet, the standard deviation of the means presented suggest that the diverse locations where measurements were taken (farms with outdoor, indoor, and half-outdoor locations, giving distinct wind protection and ventilation) might explain the variability. Indeed, the relationship between the measured concentration at the sampling point and the true CH_4_ concentration at the exhalation point is weakened by higher variability in the dilution that occurs in animals’ breath after exhalation [24]. In turn, measurements performed in respiration calorimetry chambers retrieved higher mean CH_4_ concentrations of 396 ± 182.7 and 417 ± 104.7 ppm [14,25]. Those higher CH_4_ concentrations are likely related to the natural ventilation, absent in the gas chambers, which promotes dissipation of the breath and eructed gas column, decreasing the accumulation of CH_4_. It is, thus, important that measurements with the LMD are performed under similar conditions to allow the data to be comparable. This technical requirement is possibly the most challenging to fulfill, without which data comparisons are compromised. The creation of a consensus protocol for LMD use already has been suggested to address inter-studies reproducibility and repeatability issues [15]. Nevertheless, as pointed out by other authors, LMD allows for designing a cheaper and simpler trial to monitor CH_4_ emissions compared to other currently available methods, having a great impact in financially challenged areas [22].

The results of the present study highlighted differences in daily CH_4_ emission between Jersey and Holstein cows, mainly associated with a higher concentration of ruminal CH_4_ in eructating events. Previous studies evaluating daily CH_4_ emission with respiratory chambers showed that daily emissions were higher in Holstein cows, yet when expressed as a percentage of gross energy intake, opposite results were observed [26]. Moreover, Holsteins responded to high-concentrate diets with a more pronounced decline of CH_4_ production than Jerseys [26,27], which reflects breed-related differences in ruminal fermentation patterns, likely justified by the anatomical and physiological particularities of their digestive system [28], as well as differences in the expression of genes involved in energy homeostasis [29]. Moreover, the ruminal microbiota is known to affect CH_4_ emission with bacteria providing substrates through feed degradation that methanogens utilize for methanogenesis [30]. Indeed, previous studies reported breed-related differences in the ruminal microorganisms of Jersey and Holstein cows housed together and kept under the same feeding regimen [31,32]. Differences in bacterial communities correlated with CH_4_ production, whereas the structure of the methanogen communities did not [33]. 

Winter calving is a common practice in the Azores, as it eases operations and allows profit maximization due to feeding abundance in early and peak lactation. Therefore, most cows were at early (1–90 d postpartum) or mid-lactation (91–210 d postpartum) stages at the beginning of this study. The milk traits of the dairy cows participating in the study were in line with previous studies that reported higher milk production of Holsteins with a lower concentration of both protein and fat than of Jerseys in grazing conditions [28,34]. A greater digestibility of grass likely renders Jersey cows more energy-available for milk solids production [35]. Moreover, our results agree with the knowledge that early lactation is characterized by higher milk yield, as in this stage, the peak is often reached, followed by a gradual decline, which is more pronounced at the later stage [36]. Furthermore, primiparous cows’ milk yields were lower compared to the multiparous herd fellows, which is likely explained by their lower feed intake and higher energy demand, as well as physical stress due to hierarchy fighting, gestation, and lactation [37]. When normalizing for milk yield, the daily emission of CH_4_ ceased to be significant between Holstein and Jersey cows. However, in addition to milk yield, milk nutrient density is a trait that deserves to be included in the evaluation of dairy breed-related milk sustainable production. A fine example is the work by Capper and Cady (2012), which compared the environmental impact of producing sufficient milk from either the Jersey or Holstein populations to obtain the same amount of Cheddar cheese [38]. The authors concluded that although a compensation of the Jersey population size was required to compensate for Holsteins’ higher milk yield, Jerseys’ higher milk energy relative to metabolic body weight renders more sustainable milk, with less cropland use, water consumption, nitrogen and phosphorus excretion [38]. The seemingly greater digestibility of fibers by Jersey and the efficiency per kg of live weight reinforces their suitability for pasture-based systems. This is quite relevant for the Azorean dairy industry, which still benefits from production systems closely linked to the natural environment and its resources [21], although the way subsidies are applied still benefits milk yield, rendering Holstein breeding higher profitability for farmers.

Although Azorean pastures allow year-round grazing, during the lean period—typically in the dry season (August and September) and winter [39]—silages or concentrates are offered to cover the supplemental energy expenditure of the second and third trimesters of pregnancy. As such, the feed supplementation of the herd participating in this study accounted for the parity and lactation stages. Unsurprisingly, a reflection of the dietary management of the cows’ CH_4_ emissions was found. Primiparous cows in early lactation that were supplemented with concentrate at a higher level had lower CH_4_ emissions compared to their herd fellows. The effect of the lactation stage was significant when CH_4_ emission was expressed per milk yield, which occurs since the proportion of available energy utilized for lactation is the highest in early lactation, being thereafter channeled for the reconstitution of body reserves over lactation [40]. Despite variation in their composition, concentrates are sources of high fermentable carbohydrates that naturally promote degradation through the propionate pathway, shifting, to some extent, the products available for methanogenesis [30]. However, risks associated with concentrate-rich diets include ruminal acidosis (reduction in ruminal pH), due to an imbalance in rumen fermentation rate against rumen absorption and buffering rates [41]. Particularly, subacute ruminal acidosis is a chronic disease that impairs animal health, welfare, and subsequently, milk production, while perpetuating high economic losses [41]. Moreover, a CH_4_ emission breed-associated response to high concentrate diets has been reported, with Holsteins more effective in reducing production compared to Jersey cows [26,27]. The carbon footprint associated with the concentrate feeding level has been a high priority. A study comparing two breeds (Alpine Grey and Brown Swiss) supplemented at two levels of concentrate reported that enteric CH_4_ was the most impacting factor for carbon footprint, and that its reduction was associated with high-concentrate diets [42]. The study also reported that the reduction in diversity loss and increase in carbon sequestration was linked to low-concentrate diets while acknowledging differences in diet response associated with the breed [42]. In another study, the carbon footprint of milk in a grass-based system was reported to be 5–7% lower than in confinement systems, due to carbon sequestration [43]. A study that employed life cycle assessment claimed that the grazing dairy production system in the Azores was more sustainable compared to other regions with 32% less GHG emissions [44]; however, as most studies stressed, a lack of consistency in results related to methodological choice limits concrete and objective conclusions. 

## 5. Conclusions

Our measurements of CH_4_ emissions with LMD highlighted differences related to the breed and lactation cycle in dairy cows raised under the typical Azorean semi-extensive system. These findings challenged us to reconsider the best breeding and dietary management choices to prevent higher CH_4_ emissions observed during the mid and late lactation stages. The results and protocol herein described can be employed to design and validate regional tailored measures, including dietary management to mitigate enteric methane emissions without compromising productivity. Moreover, the data obtained are relevant for geographical areas such as New Zealand or Ireland, where similar dairy breeding systems are common. Among the portable and minimally invasive equipment available for CH_4_ measurement, LMD is probably the most affordable, rendering suitability for a more widespread use on farms, for instance, as part of GHG emission monitoring programs. Recommendations for the use of LMD at the farm or cooperative level include the selection of replacement heifers, dietary changes (in response to market impositions or climate events), and the transition into organic farming. 

## Figures and Tables

**Table 1:** Descriptive statistics of the CH_4_ profiles obtained from the 55 dairy cows participating in the study.

Variables	Mean	sd	Lowest	Max
	CH_4_ Measured Values (ppm) ^b^			
SUM_CH_4_ intensity ^a^	9655	3613.2	2503	21,478
MEAN_CH_4_ intensity	80.5	30.11	20.9	179
SUM_R_CH_4_ intensity ^a^	5178	2224.3	1469	13,778
R_CH_4_ intensity	49.0	21.35	13.70	129
Respiratory peaks ^a^	22.1	3.71	13.3	32.9
SUM_E_CH_4_ intensity ^a^	4476	1655	711	10,911
E_CH_4_ intensity	337	107.1	59.0	650
% of respiratory emissions	52.6	6.65	37	72
% of eructation emissions	47.4	6.65	28	63
Eructation events ^a^	1.0	0.30	0.43	2.2

Intensity corresponds to the peak values. sd, standard deviation; max, maximum; min, minute; SUM_CH_4_, sum of all peak values; MEAN_CH_4_, arithmetic mean of all peak values; SUM_R_CH_4_, sum of respiratory peak values; R_CH_4_, arithmetic mean of respiratory peak values; SUM_E_CH_4_, sum of eructation peak values; E_CH_4_, arithmetic mean of eructation peak values. ^a^ per minute. ^b^ ppm-m was converted to ppm dividing it by the measurement distance (1.2 m).

**Table 2:** Least squares means and standard error (se) of milk and CH_4_ traits, according to breed, lactation stage, and parity of the 460 profiles.

	Breed				Lactation Stage						Parity					
	Jersey	Holstein	se	*p*	Dry	Early	Mid	Late	se	*p*	1	2	3	4	se	*p*
Milk Traits																
Milk yield (L d^−1^)	26.5	31.8	0.67	<0.001	-	32.6 ^c^	29.6 ^b^	25.3 ^a^	0.89	<0.001	24.1 ^a^	30.1 ^b^	29.0 ^b^	33.4 ^c^	1.08	<0.001
Milk fat (%)	4.70	4.16	0.084	<0.001	-	4.32 ^a^	4.21 ^a^	4.76 ^b^	0.110	<0.001	4.60	4.55	4.36	4.23	0.138	0.171
Milk protein (%)	3.88	3.31	0.037	<0.001	-	3.38 ^a^	3.54 ^a^	3.87 ^b^	0.049	<0.001	3.75 ^b^	3.61 ^a^	3.51 ^a^	3.51 ^a^	0.061	0.002
**CH_4_ traits**																
E_CH_4_ (ppm)	295	344	9.5	0.004	267 ^a^	311 ^b^	354 ^c^	348 ^c^	12.4	<0.001	290	334	319	335	16.0	0.074
R_CH_4_ (ppm)	43.1	48.9	1.75	0.059	31.8 ^a^	47.4 ^b^	54.1 ^b^	50.7 ^b^	2.26	<0.001	37.9 ^a^	47.6 ^b^	49.5 ^b^	48.9 ^b^	3.05	0.004
Eructation events	1.1	1.1	0.03	0.834	1.0	1.1	1.1	1.1	0.03	0.308	1.1	1.1	1.0	1.1	0.05	0.643
CH_4_ (g d^−1^)	53.9	60.7	2.06	0.060	43.4 ^a^	57.4 ^b^	63.9 ^b^	64.4 ^b^	2.62	<0.001	50.4 ^a^	57.5 ^ab^	57.1 ^ab^	64.1 ^b^	3.58	0.036
CH_4_:milk ^d^	2.33	2.36	0.131	0.881	-	1.79 ^a^	2.40 ^b^	2.84 ^c^	0.146	<0.001	2.48	2.16	2.40	2.34	0.212	0.586

^a–c^ Means within a row with different superscripts differ (*p* < 0.05). ^d^ g of daily CH_4_ emitted divided per daily milk yield (L).

## Data Availability

Not applicable.

## Supplementary Materials

The following supporting information can be downloaded at: https://www.mdpi.com/article/10.3390/ani13060974/s1, Table S1: Detailed characterization of the dairy cows participating in the study; Table S2: The chemical composition of the concentrate fed to dairy cows analyzed according to AOAC official methods [45], and Goering and Van Soest [46].

## References

[1] Wójcik-Gront E. (2020). Analysis of Sources and Trends in Agricultural GHG Emissions from Annex I Countries. Atmosphere.

[2] Oreggioni G.D., Monforti Ferraio F., Crippa M., Muntean M., Schaaf E., Guizzardi D., Solazzo E., Duerr M., Perry M., Vignati E. (2021). Climate Change in a Changing World: Socio-Economic and Technological Transitions, Regulatory Frameworks and Trends on Global Greenhouse Gas Emissions from EDGAR v.5.0. Glob. Environ. Chang..

[3] Bittante G., Cipolat-Gotet C., Cecchinato A. (2020). Genetic Parameters of Different FTIR-Enabled Phenotyping Tools Derived from Milk Fatty Acid Profile for Reducing Enteric Methane Emissions in Dairy Cattle. Animals.

[4] Alemu A.W., Pekrul L.K.D., Shreck A.L., Booker C.W., McGinn S.M., Kindermann M., Beauchemin K.A. (2021). 3-Nitrooxypropanol Decreased Enteric Methane Production From Growing Beef Cattle in a Commercial Feedlot: Implications for Sustainable Beef Cattle Production. Front. Anim. Sci..

[5] Baca-González V., Asensio-Calavia P., González-Acosta S., de la Lastra J.M.P., de la Nuez A.M. (2020). Are Vaccines the Solution for Methane Emissions from Ruminants? A Systematic Review. Vaccines.

[6] Danielsson R., Ramin M., Bertilsson J., Lund P., Huhtanen P. (2017). Evaluation of a Gas in Vitro System for Predicting Methane Production in Vivo. J. Dairy Sci..

[7] Garnsworthy P.C., Difford G.F., Bell M.J., Bayat A.R., Huhtanen P., Kuhla B., Lassen J., Peiren N., Pszczola M., Sorg D. (2019). Comparison of Methods to Measure Methane for Use in Genetic Evaluation of Dairy Cattle. Animals.

[8] Muñoz C., Yan T., Wills D.A., Murray S., Gordon A.W. (2012). Comparison of the Sulfur Hexafluoride Tracer and Respiration Chamber Techniques for Estimating Methane Emissions and Correction for Rectum Methane Output from Dairy Cows. J. Dairy Sci..

[9] McGinn S.M., Coulombe J.-F., Beauchemin K.A. (2021). Technical Note: Validation of the GreenFeed System for Measuring Enteric Gas Emissions from Cattle. J. Anim. Sci..

[10] Bekele W., Guinguina A., Zegeye A., Simachew A., Ramin M. (2022). Contemporary Methods of Measuring and Estimating Methane Emission from Ruminants. Methane.

[11] Lassen J., Løvendahl P., Madsen J. (2012). Accuracy of Noninvasive Breath Methane Measurements Using Fourier Transform Infrared Methods on Individual Cows. J. Dairy Sci..

[12] Rey J., Atxaerandio R., Ruiz R., Ugarte E., González-Recio O., Garcia-Rodriguez A., Goiri I. (2019). Comparison Between Non-Invasive Methane Measurement Techniques in Cattle. Animals.

[13] Sorg D., Difford G.F., Mühlbach S., Kuhla B., Swalve H.H., Lassen J., Strabel T., Pszczola M. (2018). Comparison of a Laser Methane Detector with the GreenFeed and Two Breath Analysers for On-Farm Measurements of Methane Emissions from Dairy Cows. Comput. Electron. Agric..

[14] Chagunda M.G.G., Ross D., Rooke J., Yan T., Douglas J.-L., Poret L., McEwan N.R., Teeranavattanakul P., Roberts D.J. (2013). Measurement of Enteric Methane from Ruminants Using a Hand-Held Laser Methane Detector. Acta Agric. Scand. Sect. A Anim. Sci..

[15] Sorg D. (2021). Measuring Livestock CH4 Emissions with the Laser Methane Detector: A Review. Methane.

[16] de Azevedo E.B., Centro de Estudos do Clima, Meteorologia e Mudanças Globais (2013). Cartografia Do Clima Normal Do Arquipélago Dos Açores—Ilha Terceira—Apuramento Anual—Modelo CIELO.

[17] Lanzoni L., Chagunda M.G.G., Fusaro I., Chincarini M., Giammarco M., Atzori A.S., Podaliri M., Vignola G. (2022). Assessment of Seasonal Variation in Methane Emissions of Mediterranean Buffaloes Using a Laser Methane Detector. Animals.

[18] Arnau J., Bendayan R., Blanca M.J., Bono R. (2014). Should We Rely on the Kenward-Roger Approximation When Using Linear Mixed Models If the Groups Have Different Distributions?. Br. J. Math. Stat. Psychol..

[19] SREA (2021). Serviço Regional de Estatística Dos Açores—Séries Longas Agricultura Pecuária Pescas.

[20] de Almeida A.M., Alvarenga P., Fangueiro D. (2021). The Dairy Sector in the Azores Islands: Possibilities and Main Constraints towards Increased Added Value. Trop. Anim. Health Prod..

[21] Medeiros I., Fernandez-Novo A., Astiz S., Simões J. (2021). Production and Health Management from Grazing to Confinement Systems of Largest Dairy Bovine Farms in Azores: A Farmers’ Perspective. Animals.

[22] Kobayashi N., Hou F., Tsunekawa A., Yan T., Tegegne F., Tassew A., Mekuriaw Y., Mekuriaw S., Hunegnaw B., Mekonnen W. (2021). Laser Methane Detector-Based Quantification of Methane Emissions from Indoor-Fed Fogera Dairy Cows. Anim. Biosci..

[23] Pinto A., Yin T., Reichenbach M., Bhatta R., Malik P.K., Schlecht E., König S. (2020). Enteric Methane Emissions of Dairy Cattle Considering Breed Composition, Pasture Management, Housing Conditions and Feeding Characteristics along a Rural-Urban Gradient in a Rising Megacity. Agriculture.

[24] Wu L., Koerkamp P.W.G.G., Ogink N. (2018). Uncertainty Assessment of the Breath Methane Concentration Method to Determine Methane Production of Dairy Cows. J. Dairy Sci..

[25] Chagunda M.G.G., Yan T. (2011). Do Methane Measurements from a Laser Detector and an Indirect Open-Circuit Respiration Calorimetric Chamber Agree Sufficiently Closely?. Anim. Feed Sci. Technol..

[26] Olijhoek D.W., Løvendahl P., Lassen J., Hellwing A.L.F., Höglund J.K., Weisbjerg M.R., Noel S.J., McLean F., Højberg O., Lund P. (2018). Methane Production, Rumen Fermentation, and Diet Digestibility of Holstein and Jersey Dairy Cows Being Divergent in Residual Feed Intake and Fed at 2 Forage-to-Concentrate Ratios. J. Dairy Sci..

[27] Olijhoek D.W., Hellwing A.L.F., Noel S.J., Lund P., Larsen M., Weisbjerg M.R., Børsting C.F. (2022). Feeding up to 91% Concentrate to Holstein and Jersey Dairy Cows: Effects on Enteric Methane Emission, Rumen Fermentation and Bacterial Community, Digestibility, Production, and Feeding Behavior. J. Dairy Sci..

[28] Aikman P.C., Reynolds C.K., Beever D.E. (2008). Diet Digestibility, Rate of Passage, and Eating and Rumination Behavior of Jersey and Holstein Cows. J. Dairy Sci..

[29] Alam T., Kenny D.A., Sweeney T., Buckley F., Prendiville R., McGee M., Waters S.M. (2012). Expression of Genes Involved in Energy Homeostasis in the Duodenum and Liver of Holstein-Friesian and Jersey Cows and Their F_1_ Hybrid. Physiol. Genom..

[30] Pereira A.M., de Lurdes Nunes Enes Dapkevicius M., Borba A.E.S. (2022). Alternative Pathways for Hydrogen Sink Originated from the Ruminal Fermentation of Carbohydrates: Which Microorganisms Are Involved in Lowering Methane Emission?. Anim. Microbiome.

[31] Paz H.A., Anderson C.L., Muller M.J., Kononoff P.J., Fernando S.C. (2016). Rumen Bacterial Community Composition in Holstein and Jersey Cows Is Different under Same Dietary Condition and Is Not Affected by Sampling Method. Front. Microbiol..

[32] King E.E., Smith R.P., St-Pierre B., Wright A.-D.G. (2011). Differences in the Rumen Methanogen Populations of Lactating Jersey and Holstein Dairy Cows under the Same Diet Regimen. Appl. Environ. Microbiol..

[33] Noel S.J., Olijhoek D.W., Mclean F., Løvendahl P., Lund P., Højberg O. (2019). Rumen and Fecal Microbial Community Structure of Holstein and Jersey Dairy Cows as Affected by Breed, Diet, and Residual Feed Intake. Animals.

[34] Palladino R.A., Buckley F., Prendiville R., Murphy J.J., Callan J., Kenny D.A. (2010). A Comparison between Holstein-Friesian and Jersey Dairy Cows and Their F1 Hybrid on Milk Fatty Acid Composition under Grazing Conditions. J. Dairy Sci..

[35] Beecher M., Buckley F., Waters S.M., Boland T.M., Enriquez-Hidalgo D., Deighton M.H., O’Donovan M., Lewis E. (2014). Gastrointestinal Tract Size, Total-Tract Digestibility, and Rumen Microflora in Different Dairy Cow Genotypes. J. Dairy Sci..

[36] Vijayakumar M., Park J.H., Ki K.S., Lim D.H., Kim S.B., Park S.M., Jeong H.Y., Park B.Y., Kim T. (2017). Il The Effect of Lactation Number, Stage, Length, and Milking Frequency on Milk Yield in Korean Holstein Dairy Cows Using Automatic Milking System. Asian-Australas. J. Anim. Sci..

[37] Walter L.L., Gärtner T., Gernand E., Wehrend A., Donat K. (2022). Effects of Parity and Stage of Lactation on Trend and Variability of Metabolic Markers in Dairy Cows. Animals.

[38] Capper J.L., Cady R.A. (2012). A Comparison of the Environmental Impact of Jersey Compared with Holstein Milk for Cheese Production. J. Dairy Sci..

[39] Melo C.D., Maduro Dias C.S.A.M., Wallon S., Borba A.E.S., Madruga J., Borges P.A.V., Ferreira M.T., Elias R.B. (2022). Influence of Climate Variability and Soil Fertility on the Forage Quality and Productivity in Azorean Pastures. Agriculture.

[40] Prendiville R., Pierce K.M., Delaby L., Buckley F. (2011). Animal Performance and Production Efficiencies of Holstein-Friesian, Jersey and Jersey × Holstein-Friesian Cows throughout Lactation. Livest. Sci..

[41] Elmhadi M.E., Ali D.K., Khogali M.K., Wang H. (2022). Subacute Ruminal Acidosis in Dairy Herds: Microbiological and Nutritional Causes, Consequences, and Prevention Strategies. Anim. Nutr..

[42] Sabia E., Kühl S., Flach L., Lambertz C., Gauly M. (2020). Effect of Feed Concentrate Intake on the Environmental Impact of Dairy Cows in an Alpine Mountain Region Including Soil Carbon Sequestration and Effect on Biodiversity. Sustainability.

[43] O’Brien D., Capper J.L., Garnsworthy P.C., Grainger C., Shalloo L. (2014). A Case Study of the Carbon Footprint of Milk from High-Performing Confinement and Grass-Based Dairy Farms. J. Dairy Sci..

[44] Morais T.G., Teixeira R.F.M., Rodrigues N.R., Domingos T. (2018). Carbon Footprint of Milk from Pasture-Based Dairy Farms in Azores, Portugal. Sustainability.

[45] AOAC (2000). Official Methods of Analysis. Association of Official Analytical Chemists.

[46] Van Soest P.J., Robertson J.B., Lewis B.A. (1991). Methods for Dietary Fiber, Neutral Detergent Fiber, and Nonstarch Polysaccharides in Relation to Animal Nutrition. J. Dairy Sci..

