# Supplementary material for: A Longitudinal Study with a Laser Methane Detector (LMD) Highlighting Lactation Cycle-Related Differences in Methane Emissions from Dairy Cows
Source: Animals (Basel). 2023 Mar 8;13(6):974. doi: 10.3390/ani13060974 (PMC10044636; doi:10.3390/ani13060974)
Supplement: Supplementary file 1 [file animals-13-00974-s001.zip › animals-2231710-SI.pdf]

Supplementary material

Table S1. Detailed characterization of the dairy cows participating in the study

	Time points									
	1	2	3	4	5	6	7	8	9	10
**Total cows**										
	36	37	39	43	49	48	51	52	52	53
**Breed**										
Jersey	8	8	8	10	10	11	12	13	12	12
Holstein	28	29	31	33	39	37	39	39	40	41
**Lactation stage**										
Dry	4	4	5	9	15	5	2	2	3	5
Early	13	14	11	10	10	18	22	21	20	20
Mid	11	11	15	16	17	12	12	13	12	13
Late	8	8	8	8	7	13	15	16	17	15
**Parity**										
1	14	14	14	14	15	15	16	15	15	16
2	7	10	10	11	12	15	13	15	14	14
3	10	8	10	12	14	11	14	14	15	15
≥4	5	5	5	6	8	7	8	8	8	8

Table S2. The chemical composition of the concentrate fed to dairy cows analyzed according to AOAC official methods [45] and Goering and Van Soest [46](Van Soest. Robertson. and Lewis 1991)

	CP	NDF	ADF	ADL	EE	Ash	DM
**%**	21.7	37.5	9.67	3.29	5.48	9.58	85.5
**Method**	AOAC 954.01	Goering and Van Soest			AOAC 920.39	AOAC 942.05	AOAC 930.15

CP, crude protein; NDF, neutral detergent fiber; ADF, acid detergent fiber; ADL, acid detergent lignin; EE, ether extract; DM, dry matter
